# Exploring mental health challenges encountered by educators in the South Asian countries—a qualitative survey

**DOI:** 10.3389/fpsyg.2026.1682410

**Published:** 2026-04-30

**Authors:** Anjum Bano Kazimi, Munir Moosa Sadruddin, Sohni Siddiqui, Mevlut Aydogmus

**Affiliations:** 1 Department of Educational Sciences, Iqra University, Karachi, Pakistan; 2 Department of Education, SMI University, Karachi, Pakistan; 3 Institute for Educational Research, Bergische Universität Wuppertal, Wuppertal, Germany; 4 Department of Curriculum and Instruction, Necmettin Erbakan University, Konya, Türkiye

**Keywords:** cultural stigmatization, school education, South Asian countries, teacher mental health and well-being, thematic analysis

## Abstract

**Introduction:**

Mental health challenges are a growing concern across academia, significantly impacting teachers’ well-being and, ultimately, the quality of education provided to students. This study aims to understand mental health challenges encountered by teachers in their workplace and employs an online qualitative survey where online interviews with three semi-structured questions with large-scale data set of 700 teachers conducted to collect data.

**Methods:**

Participants were school teachers, selected via quota sampling from seven South Asian countries (Pakistan, India, Sri Lanka, the Maldives, Bhutan, Afghanistan and Nepal) through a virtual global teaching platform. The qualitative data were analysed using thematic analysis, beginning with open coding to facilitate initial conceptualisation. This was followed by axial coding, in which the research team organised the primary codes into coherent sub-themes. To ensure investigator triangulation and mitigate individual biases, multiple experts collaborated during the analysis to enhance the analytical rigour of the final themes.

**Results:**

Despite a diversity of contexts worldwide, the findings confirm that the challenges faced by teachers in all seven developing countries share commonalities: burnout and excessive workload, lack of communication and exclusion from decision making, exploitation, professional jealousy and toxic work place. The barriers to discussing mental health issues include a culture that discourages sharing, a lack of awareness, and the unavailability of mental health services in the workplace. To cope, teachers often rely on sharing their feelings with friends and family, practicing meditation, engaging in recreational activities, improving communication, and participating in community engagement.

**Discussion:**

It is recommended to introduce mental health policies in the workplace parallel with empowering teachers to enhance their well-being through self-empowerment courses and counseling. Further, there is a dire need to establish a mental health services unit for teachers.

## 1 Introduction

Teachers play a significant role in the lives of learners and the productivity of institutions as a whole. Their identity is dynamic and multifaceted, comprising in-depth narratives, values, emotions and a sense of self within the teaching profession (Craig, 2023). All of these evolve with experience, exposure and interactions. Teaching is a challenging profession due to its professional complexities, standardized performativity and increasing accountability (Siddiqui et al., 2023a). Teachers are expected to maintain healthy relationships at work while balancing personal and professional responsibilities, which can be challenging when the workplace environment is not conducive (Shah et al., 2024; Siddiqui et al., 2025) or when expectations are not met. If unaddressed over time, these challenges can lead to mental health issues and affect teachers’ overall well-being (Bernotaite and Malinauskiene, 2017).

According to the World Health Organization (WHO), mental health is defined as “a state of well-being in which an individual realizes their own potential, can cope with the normal stresses of life, can work productively and fruitfully, and can contribute to their community” [World Health Organization (WHO), 2024]. Well-being reflects an individual’s overall satisfaction and is shaped by interconnected factors such as spirituality, financial stability, social circumstances and psychological well-being (Jordan, 2023). This multidimensional concept is a key topic in policy discussions due to its strong association with work productivity, effective learning and quality of life. However, clear conceptualization and operational definition of well-being remain elusive.

Positive well-being is associated with personal, professional, and interpersonal success. Studies confirm that individuals with positive well-being and strong social connections perform well in social, academic, and professional relationships, and send positive vibes to their surroundings (for e.g., Jordan, 2023; Lyubomirsky et al., 2005; Ruggeri et al., 2020; Torres-Soto et al., 2022; Umberson and Montez, 2010).

Global organizations have highlighted the importance of workplace wellbeing. The World Health Organization and the International Labor Organization have emphasized the importance of promoting well-being and protecting mental health in the workplace, and of providing support to individuals with mental health conditions [International Labour Organization, 2009; World Health Organization (WHO), 2024]. Similarly, Sustainable Development Goal 3 aims to achieve good health and well-being for all by 2030 (Sustainable Development Goals, 2023). While this goal primarily emphasizes reducing mortality, combatting communicable diseases and preventing substance abuse, it also plays a crucial role in addressing mental health issues. Likewise, the United Nations System Mental Health and Well-being Strategy focuses on ‘creating an inclusive, sustainable work environment where mental health and well-being are embedded in organizational culture and systems—where everyone belongs, is valued, nurtured, and thrives, ensuring an efficient workforce’ (United Nations, 2024, p. 2). A good working environment, fair salary, and positive social relations play a significant role in the life of an employee. Conversely, a negative working environment, peer pressure, job insecurity, and other stressors significantly impact workplace output and well-being (Gedikli et al., 2023; Gu et al., 2022; Ray, 2022; Waddell and Burton, 2006).

The teaching profession is currently facing a severe shortage of staff, largely due to poor working conditions, a low social status, a low salary, a lack of autonomy and an excessive workload (Siddiqui et al., 2023a; UNESCO, 2023a). One report highlighted that around 44 million additional teachers will be required by 2030 to meet the targets for primary and secondary education (UNESCO and International Task Force on Teachers for Education, 2024). A key issue that remains under-researched and under-practiced, particularly in developing countries, is addressing the mental health and wellbeing of teachers. Amid the rising demand for educators and a simultaneous labor shortage, it is important to consider why many individuals are reluctant to pursue a career in teaching. What motivates individuals to become teachers, and what causes them to leave when other opportunities arise? Understanding the challenges that teachers face is essential to creating a supportive environment that encourages retention and inspires more individuals to enter the teaching profession with confidence.

This study aims to identify the mental health issues faced by teachers in developing countries, and to establish the primary causes of poor mental well-being. This study is novel in that it is the first to explore a substantial dataset encompassing seven South Asian nations that are members of SAARC. These countries share many similarities, including a colonial history, linguistic links, culture and cuisine, as well as complex socio-economic challenges, particularly the significant stigma surrounding mental health (Dua et al., 2023; Vaishnav et al., 2023). Unfortunately, amid major societal struggles, especially the pursuit of financial stability, core mental health issues are often overlooked and stigmatized. Unlike in developed countries, where support models and networks for mental health are in place, this region struggles to recognize the importance of mental well-being. In this context, the study goes beyond merely assessing the prevalence of mental health issues or conducting cross-sectional surveys that connect different variables, as has been seen in previous research (Aryal et al., 2025; Dorji et al., 2019; Javed et al., 2021; Kalam et al., 2024; Miri and Timori, 2021; Ray and Guha, 2025; Senevirathne et al., 2025). Instead, it aims to identify the root causes and propose solutions to help teachers maintain their well-being. The findings of this study will enable educators, administrators and policymakers to identify the underlying causes of mental health issues among teachers and develop strategies to promote their well-being and retention in this noble profession.

### 1.1 Research questions

1. What are the most common mental health challenges faced by teachers at work?
2. What barriers prevent teachers from seeking mental health support?
3. What are the best practices used by teachers to promote their mental health?

## 2 Literature review

The well-being of teachers is paramount to fostering a positive environment within educational institutions (Hascher and Waber, 2021). Teacher well-being encompasses more than just the absence of illness; it signifies educators’ healthy and successful performance in their professional roles. The issue has received considerable attention in recent decades due to a significant rise in teachers taking sick leave or leaving their jobs globally (Benevene et al., 2020). Specially, the COVID-19 pandemic significantly impacted school teachers’ psychological well-being. The combined stress of an increased workload, the need to manage multiple tasks, negative job perceptions, concern for others’ welfare, personal health struggles, and the requirement to play multiple roles collectively damaged their mental health and well-being (Kim et al., 2022). There is a consensus among educators and researchers that teaching is an inherently stressful occupation, causing teachers to experience mental health problems at a higher rate than in many other professions (Kinman et al., 2011). International research consistently highlights the high prevalence of mental health issues within the teaching profession. A scoping review by Agyapong et al. (2022) found that globally, teachers reported moderate-to-severe psychological conditions, with prevalence rates for burnout ranging from 25.12 to 74%, stress from 8.3 to 87.1%, anxiety from 38 to 41.2%, and depression from 4 to 77%. In support of this, a study of Italian teachers found that around half exceeded the clinical threshold for depression and approximately one in 10 met the criteria for anxiety, as determined by self-reporting measures. This poor mental health is strongly correlated with a combination of high job demands and low social support (Borrelli et al., 2014). These high rates are driven by specific occupational stressors. For instance, a study of Norwegian teachers by Skaalvik and Skaalvik (2017) demonstrated that while multiple factors contribute to emotional exhaustion, time pressure exerted the strongest effect. Similarly, Betoret (2009) linked emotional exhaustion resulting from both internal and external job stressors to the high rates of burnout observed among Spanish school teachers.

Recent studies confirm that teachers’ well-being directly influences their classroom practices, particularly the quality of teaching, staff retention and student well-being, which eventually improves learning and academic outcomes (Bilz et al., 2022; Dreer, 2023; Harding et al., 2019; Li et al., 2022; McCallum, 2021). A positive sense of well-being is strongly associated with teachers’ willingness to support learners facing mental health challenges as well (Turner and Thielking, 2019).

Teachers’ well-being is influenced by various factors, including the quality of trust relationships with colleagues (Hascher and Waber, 2021), effective leadership practices (Collie et al., 2020), personal capabilities (Collie et al., 2020), socio-emotional competence (Collie, 2025), workplace recognition (Yahya and Azeez, 2024), job security (Özmen et al., 2025), positive student–teacher relationships (Forster et al., 2022), occupational commitment (Collie et al., 2020), motivation (Collie et al., 2020), hope (Collie et al., 2020), continuous professional development (Collie et al., 2020) and healthy working relationships (Hascher and Waber, 2021). In addition, Sewani (2023) designed a teacher identity model and emphasized that it helps teachers understand their identity and supports their well-being. He stated, “teachers should reflect on their personal experiences and explore their insecurities, which they tend to keep in their subconsciousness. This could be detrimental to their self-acceptance, and it could also affect their teaching. By exploring their personal identity, teachers can improve their mental well-being, better understand themselves, and become more effective teachers.”

When school leadership is positive and a sense of fairness and equity is practiced in the workplace, a peer-to-peer support system is available and teachers’ self-efficacy improves (Collie et al., 2020). Conversely, factors such as external pressures, lack of recognition, over-expectations, turnover intentions, professional burnout, lack of digital literacy, limited career advancement opportunities, lack of a supportive system and a toxic workplace environment can negatively impact teachers’ well-being (Education and Solidarity Network and the Foundation for Public Health, 2023; Ghamrawi et al., 2023; Hascher and Waber, 2021; Nwoko et al., 2023; Siddiqui et al., 2023a; Tsuyuguchi, 2023; University of Oxford, 2024; Viac and Fraser, 2020; Zhou et al., 2024). Similarly, negative student behavior can have a detrimental impact on teachers’ professional and personal self-esteem (Forster et al., 2022; Siddiqui et al., 2025; Spilt et al., 2011).

The rationale for considering South Asia for this particular research is based on the fact that teachers in this continent in particular is mostly neglected due to lack of availability of programs to train teachers for their wellbeing, which is cause for concern, reflecting a confluence of challenges impacting the well-being and professional efficacy of teachers, and ultimately the quality of education in the region (Piyakun, 2023; Ratanasiripong et al., 2022). The World Bank (2018) has highlighted a skills and motivation gap among teachers in low- and middle-income countries. In addition, teachers face significant workplace pressures. The scarcity of resources and professional development opportunities negatively impacts learning outcomes (Kazmi et al., 2023; Siddiqui et al., 2023a). Another report emphasized the issue of teacher quality in Southeast Asia (UNICEF, 2018). The Global Education Monitoring Report (UNESCO, 2023b) reveals that, although almost all countries in Southeast Asia have ICT standards for teachers, many educators lack the necessary digital competence, which in turn negatively impacts teachers’ well-being (Siddiqui et al., 2023a). Addressing these challenges, and others, is crucial for improving teacher effectiveness and educational quality in the region.

Not all academic institutions have designed policies to support teachers’ mental health and well-being due to various factors, differing institutional priorities and a lack of awareness or understanding of the importance of such support (Yu et al., 2022). Institutions in South Asia mostly focus on academic performance (Ahmed et al., 2022), overlooking the critical need for initiatives to support teacher well-being. Ideally, supporting teachers’ well-being involves providing professional learning opportunities and adequate salaries, as well as offering support from employers (Wu and Lu, 2022). In developing countries, particularly in Africa, factors that contribute positively to teachers’ well-being include self-respect, feelings of accomplishment, relationships with students, and independence (UNESCO, 2023c). Issues that negatively impact teachers’ well-being include burnout, a lack of collaboration, weak incentives, job stress and a lack of social support mechanisms (Fourie and de Klerk, 2024; UNESCO, 2023c). The situation is even worse for teachers working in conflict zones (Powell et al., 2025).

There is a significant gap in the literature regarding teachers’ subjective experiences of well-being across South Asian countries. While numerous studies have been conducted across the SAARC region—including work from India (Chaudhry and Chhajer, 2023), Pakistan (Aslam et al., 2026), Sri Lanka (Fernandez-Chung and De Zoysa, 2022), Afghanistan (Murendo et al., 2024), Bhutan (Tshomo, 2025), Maldives (Hassan et al., 2024), and Nepal (Rejina, 2024)—to document the impact on teacher mental well-being, a detailed, focused analysis has been lacking. This research addresses that gap by serving as the first comprehensive study to thoroughly explore the underlying reasons and propose specific, actionable solutions for improving teacher well-being across these regions.

This study explores teachers’ lived experiences of mental health challenges and the best practices they adopt to support their well-being. Teachers’ well-being is of the utmost importance for several reasons. When teachers are mentally relaxed, they can focus more effectively on their professional development and perform better in the workplace (Hascher and Waber, 2021). A positive attitude enables them to excel and feel connected to their workplace, colleagues and students (Hascher and Waber, 2021). However, we must ask: what is the state of teachers’ well-being? Is the working environment supportive of their well-being? Teaching is often regarded as a noble profession, but do those in positions of authority truly value teachers as they deserve? This study aims to address these questions and contribute to the literature on the issues faced by teachers in South Asia that negatively affect their mental well-being.

## 3 Research methodology

The research design employs an online qualitative survey where large set of teachers (N = 700) were interviewed online. A qualitative survey is a research methodology that uses a standardized or semi-standardized data collection instrument, such as an open-ended or semi-structured questionnaire, to gather descriptive, in-depth data from a large or broad sample of participants. Unlike highly focused qualitative methods with small samples (such as phenomenology), the qualitative survey prioritizes breadth of perspective across a defined population in order to identify recurrent patterns, experiences or themes, rather than achieving deep saturation within an individual context.

Seven hundred school teachers (100 teachers from each participating country) were selected through quota sampling from seven South Asian countries: Pakistan, India, Sri Lanka, the Maldives, Bhutan, Afghanistan and Nepal. Quota sampling is a non-probability sampling technique in which the researcher first identifies the relevant categories, or strata, within a population (Nikolopoulou, 2023). In this case, the sample is school teachers with at least 10 years’ experience from a country in the SAARC region. The sampling methodology was adopted to get broader vision on mental health issues among the participating countries, requiring a standardized fixed sample size of 100 per country to ensure equal weighting and analytical consistency across the seven nations. The sample size of 700 was not chosen to achieve thematic saturation within a single population, but was predetermined using a quota-based design (100 participants per country) to ensure adequate representation for robust analysis across seven distinct geopolitical contexts. Adopting an emergent saturation criterion for sample cessation would have compromised the study’s fundamental rigor.

Teachers were invited to participate via the Facebook page of the Global Forum for Teacher Educators^1^. Invitations were sent to 22,000 teacher members, but only those from the specified countries were shortlisted. Further selection criteria included having a minimum of 10 years’ teaching experience and being currently employed at any public or private school (Refer to Table 1).

**Table 1:** Demographics.

Country	Male	Female
Pakistan	28	72
India	13	87
Sri Lanka	31	69
Maldives	40	60
Bhutan	11	89
Afghanistan	52	48
Nepal	12	88
Total	187	513

In accordance with ethical standards, a two-tier informed consent process was employed. Firstly, participants provided written consent via an electronic form distributed via the recruitment email (see Appendix 1). By selecting the “Agree” button at the commencement of the online invitation (see Appendix 1), participants explicitly consented to the study conditions. This digital consent form detailed the project’s objectives, participation prerequisites, data confidentiality protocols, and the voluntary nature of the study, including the right to withdraw at any time. Furthermore, the form outlined the subsequent steps for those selected, including the Zoom interview process, the requirement for verbal consent, and the expected duration. In accordance with ethical guidelines only those who agreed to these terms and provided the necessary demographic and contact information for pre-selection were contacted. Prior to the interviews, participants received an invitation email containing the Zoom link and the interview questions to ensure transparency and participant comfort. At the start of the interview session, verbal consent was re-obtained and recorded to ensure that all participants were fully informed and comfortable with proceeding (see Appendix 2). This stage involved reiterating the project goals, interview duration, data access policies, and confirming the absence of physical or psychological risks. Participants were invited to ask questions and were required to re-confirm their understanding of the terms before proceeding. No emotional distress was anticipated or observed, as the interview did not include any personal or sensitive questions. All the questions were validated by experts to ensure no sensitive questions are included.

Data were collected through qualitative interviews comprising three semi-structured questions (refer to section 1.1, research questions). These interviews were conducted and recorded online via Zoom between March 2023 and February 2024 and transcribed by one of the researchers in March 2024. Participants were recruited via an online professional forum, of which the second author is a voluntary member. Using the forum’s member directory, an initial recruitment email containing a Google Form link was distributed to assess interest and collect basic demographic data for screening purposes. Respondents who met the pre-defined inclusion criteria were then contacted via a follow-up email to schedule an interview. Once a date and time were confirmed, a secure Zoom link was provided for the session.

The data were analyzed by the first three authors of the study using thematic analysis. Thematic analysis is a widely used qualitative research method for identifying, analyzing and reporting patterns (or themes) within a dataset. These datasets are often made up of interview or focus group transcripts (Lochmiller, 2021). It is a flexible method that goes beyond simple description to interpret and explain patterns of meaning. The core objective of thematic analysis is to systematically identify recurring ideas, opinions, experiences or topics throughout the entire dataset. These themes were developed based on recurring insights, and illustrative quotes were used to ensure transparency and strengthen the validity of the findings.

The qualitative data were analyzed using thematic analysis, beginning with open coding to facilitate initial conceptualization. This was followed by axial coding, in which the research team organized the primary codes into coherent sub-themes. To ensure investigator triangulation and mitigate individual biases, multiple experts collaborated during the analysis to enhance the analytical rigor of the final themes.

## 4 Data analysis

**Figure 1:**
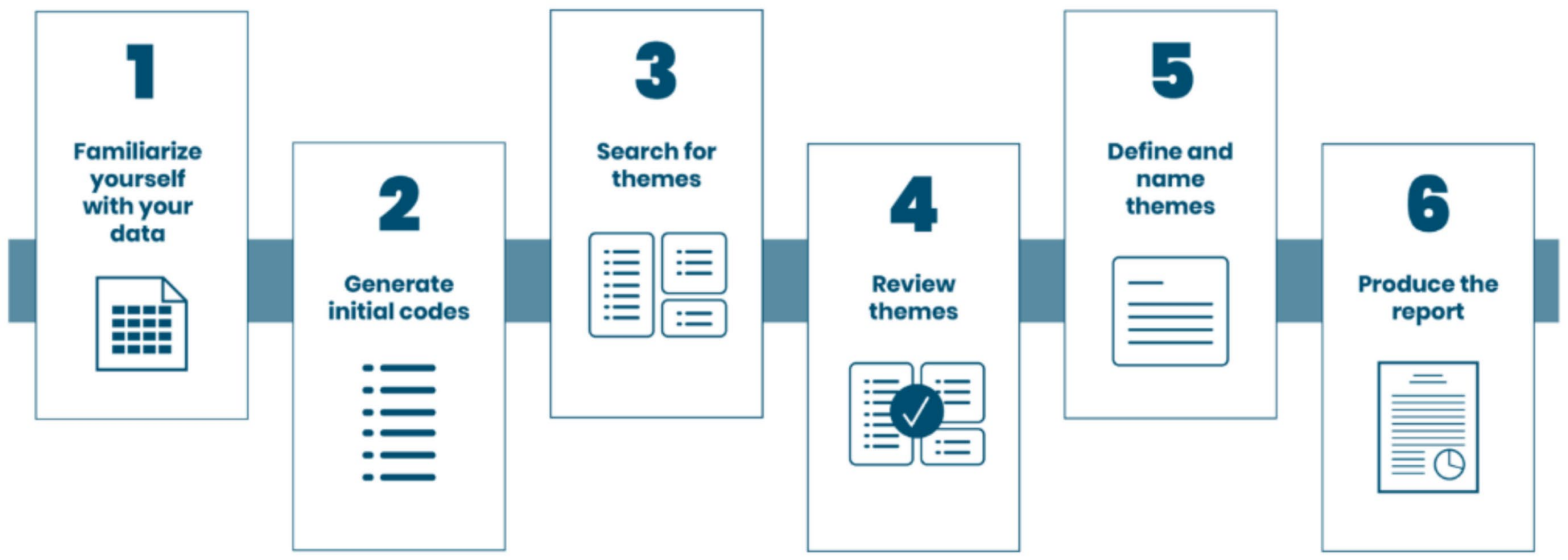
Phases of thematic analysis. Adapted from blog by Fleming (2023).

Data analysis was conducted using thematic analysis, in line with the methodology set out by Braun and Clarke (2006) (Refer to Figure 1). However, to enhance the rigor and systematic nature of our coding process, we employed techniques inspired by Strauss and Corbin’s Grounded Theory (Strauss and Corbin, 1998). Specifically, we used open coding (in phase 2, generating initial codes) for initial conceptualization and axial coding (in phase 3, searching for themes) to organize our codes into coherent sub-themes. It is important to note that the data were not managed or analyzed using qualitative analysis software such as MAXQDA, NVivo or Atlas.ti. The full analysis was instead conducted manually by the research team. Researchers prioritized trustworthiness by implementing a systematic manual analysis. A key component of this approach was investigator triangulation: three researchers independently analyzed and coded a subsample of the data, which was essential for preventing individual biases and ensuring the analytical rigor of the resulting themes.

The identified themes were then organized into three sections that aligned with the proposed research questions. To answer Research Question 1, three major sub-themes were identified. Similarly, Research Questions 2 and 3 were addressed by two major themes, neither of which included sub-themes.

### 4.1 Theme 1: common mental health challenge encountered by teachers

**Figure 2:**
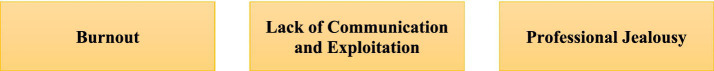
Core reasons for mental health challenges.

Teachers are expected to juggle multiple responsibilities at work, which can significantly increase their risk of experiencing well-being issues. From the data gathered through interviews, seventeen themes emerged. Three main themes were selected that resonated with more than a hundred participants (See Figure 2). Gender was not taken into consideration in selecting the final responses.

#### 4.1.1 Excess workload and burnout

When discussing the mental health challenges faced by participants, burnout was identified as a primary concern, attributed to overwhelming pressure from additional workloads and tasks (Aslam et al., 2026; Maslach and Leiter, 2016). Burnout refers to emotional, mental and physical exhaustion resulting from repetitive behaviors or situations that cause stress (Maslach and Leiter, 2016).

Participants described how unexpected additional demands from management often lead to frustration and anger as they require extra time and commitment, which has an adverse effect on their other responsibilities and personal lives. In South Asia, women are traditionally expected to be the primary caregivers and are responsible for household chores (Hinduja et al., 2023). Combined with their professional duties, this dual burden severely disrupts women’s work-life balance, often leading to burnout and exacerbating mental health issues. One female participant shared:

I wasn’t given a clear job profile when I started my job. Over time, the management recognized my capabilities and began assigning me more responsibilities without any increase in my salary. Working beyond the required hours has led to family issues, as I’m unable to spend quality time with my children and husband. I once requested the management to relieve me from additional tasks, but they turned their ear deaf to my request.

Financial and economic constraints often necessitate that many students take on employment to fund their education (Russell et al., 2025). However, the resulting additional job responsibilities place significant strain on these student-teachers, contributing to increased burnout and poor mental well-being. Participant reflected,

I experience significant stress and burnout because my boss treats me like a robot. I had hoped to pursue further qualifications alongside my job, but that hope is fading as I feel increasingly emotionally drained and unable to manage the demands of teaching.

One other participant was in view that burnout has affected his life so much that she feels drained each day and looking for a big break. Numerous studies have already documented that excessive job demands are a key factor driving teachers to leave the profession entirely (Arnold and Rahimi, 2025; Benevene et al., 2020; Siddiqui et al., 2023a).

I feel completely burned out. The endless paperwork, unrealistic demands, and lack of personal time have drained my passion for teaching. I’m just going through the motions, counting down the days until the next break. I don’t know how much longer I can keep this up.

The growing number of educators leaving the profession is largely due to the increase in administrative and non-instructional tasks beyond classroom teaching (Pressley et al., 2026). One of the participants reflected on her multiple tasks, which have contributed to her burnout.

I am a teacher, a counselor, a typist, a computer operator, a curriculum designer, a reader, a note taker, a trainer essentially, a slave. I have decided to quit this job because I can’t continue to please my boss with insincere compliments. I’ve learned how to say no, which is why I’m considered the black sheep. I would prefer giving home tuitions, where at least no one would treat me like a slave.

Many participants voiced their frustrations about the heavy workload, which makes balancing various responsibilities challenging. Some expressed concerns that their institution’s leadership fails to recognize their talents and skills, instead misusing them. Ineffective leadership is also a significant contributor to stress and burnout, and intentions to leave this profession (Bieńkowska et al., 2025; Smith and Cooper, 1994), a sentiment echoed by the participants. The frustration stemming from increased work demands placed upon teachers with advanced skills and capabilities, without corresponding financial compensation (Pressley et al., 2026) or recognition (Andina-Díaz et al., 2025), is a significant driver of burnout and a potential cause for them leaving the profession.

One participant remarked:

Is it a sin to have talent and skills? If other teachers don’t have these skills, why does the boss expect me to meet all the demands and do all the work? If the boss is incompetent, he should leave the position and should not abuse the leadership position. I was hired based on my skills, but being assigned additional tasks and pressured even after working hours is inhumane.

Another participant voiced a similar frustration, noting that the combination of high job demands and the pressure to engage in inappropriate practices, such as granting undue favors to ineligible students, created a significant ethical conflict and moral injury. Moral injury, defined as the distress caused by the transgression of core moral beliefs, has transitioned from military discourse into the educational sphere. For teachers, this injury often stems from a disconnect between their ethical obligations and the reality of institutional limitations. When systemic pressures hinder an educator’s ability to act according to moral beliefs, the resulting moral conflict frequently leads to chronic burnout (Oberg, 2025). The dilemma is compounded by the fact that the parents of these students and some staff members are often involved in enforcing these unethical practices to obtain undue favors for their children and themselves. When a teacher attempts to uphold professional standards and raises concerns, these confrontations frequently create unnecessary conflict and serious issues for the educator striving to work with dignity. For individuals committed to working with dignity and honesty, these pressures further elevate burnout and dissatisfaction.

I have been in the field of teaching for ten years. Although I enjoy my work as a teacher, there are times when I consider quitting due to stress and anxiety. The administration treats us like a donkey. They place excessive pressure on us and even ask us to give extra marks to certain students based on personal relationships. This profession no longer feels dignified. Sincere teachers are exploited, and dealing with uncooperative students, parents and colleagues only add to the difficulty.

In many professions, increased work pressure is rewarded with benefits like raises or better housing; however, teaching is known for its high workload and limited financial or supportive compensation (Schaack et al., 2022). This inequity is a key factor driving teacher frustration, burnout, and diminished mental well-being. One of the participants grieved:

We are responsible for preparing lesson plans, teaching effectively, performing well in evaluations, meeting academic standards, guiding students ethically, understanding child psychology, interacting with parents, participating in professional development, pursuing higher education, and obtaining additional certifications. Often, we work long hours and face heavy workloads, which lead to exhaustion and burnout. Meeting all the deadlines set by the administration can be challenging. They need to recognize that these tasks require time and commitment, and added pressure can compromise the quality of our work. I believe the school should adopt us and provide housing for us and our families on the premises to meet their expectations 24/7.

Burnout is a common challenge faced by teachers in various settings. Several documents have highlighted similar issues among teachers in various contexts (Agyapong et al., 2022; Madigan et al., 2023; UNESCO, 2023a, 2024). Factors contributing to teacher burnout include an excessive workload (Siddiqui et al., 2023a), a lack of administrative support (Bieńkowska et al., 2025; Smith and Cooper, 1994), insufficient resources (Kazmi et al., 2023), the pressures of high-stakes testing, challenges in classroom management (Siddiqui et al., 2023b), and the emotional demands of catering to the diverse needs of students (Kazmi et al., 2023).

Some participants who work with children who learn slowly or have attention deficit hyperactivity disorder (ADHD) believe that specialized training is necessary to manage these students effectively. However, they are often required to do so in mainstream classes without adequate support. It is important to note that while the teaching profession in many countries requires specialized training (such as a Bachelors of Education, teaching certificates or license) in addition to a subject major, these programs are often not designed to equip teachers to work with children with special needs (Alhassan et al., 2025). The increasing demand for inclusive education, integrating students with disabilities into mainstream classrooms has placed a significant burden on educators (Alhassan et al., 2025). Forcing teachers who are trained for general education to instruct students with disabilities without providing adequate specialized training is a key contributor to increased burnout and frustration (Alhassan et al., 2025). One participant shared their experience,

I have two students with ADHD in my class. I am not trained to handle their needs, and they cause disruptions that make it challenging to manage classroom discipline. I respect these students, but they require teachers with specialized training. When I requested support from the management, they threatened me with either continuing to accommodate these students or quitting. Given the financial pressures, I am forced to compromise and continue teaching under these conditions.

Providing an assistant teacher with specialized knowledge can effectively reduce the job demands on the lead teacher and ensure better individual support for students with special needs (Kamran et al., 2022). However, many schools operate under the expectation that the teacher is capable of managing everything alone, leading them to reluctantly provide additional support. This reluctance often stems from a money-making model where minimizing operational spending is prioritized over the quality of education and teacher well-being. Another participant underlined,

"I have completed a Bachelor's degree in Education, but we were not taught how to deal with slow learners. I have a few slow learners in my class who need more time and support. With fifty students in the class, I am unable to give them the extra attention they need. The management is not willing to hire a teaching assistant, just to save money."

These reflections highlight how the relentless demands, lack of support and training and exploitation of teachers in the profession contribute to burnout, affecting their personal and professional lives. Well-trained teachers are crucial for the success of all children, making investment in teacher education fundamental to delivering a quality education (Kalagbor-Gbeke, 2025). However, teacher training models in South Asia are often inadequate, failing to prepare educators for diverse classrooms and to equip them with the confidence, knowledge and skills to support learners with disabilities effectively, as Singal (2021) notes. This highlights the urgent need for appropriate initial training and continuous professional development for teachers. Furthermore, the lack of specialized training for children with special educational needs and disabilities (SEND) contributes significantly to stress and burnout among teachers in education settings (Candeias et al., 2021).

#### 4.1.2 Lack of communication and exclusion from decision making

Expressing grievances is one of the most challenging tasks in any profession, including teaching. This is because there is a general understanding that doing so may have a negative impact on one’s reputation and position in the workplace (Morrison and Milliken, 2000). The fear of negative repercussions, such as damage to one’s reputation, career prospects or relationships with superiors/colleagues, is a primary reason for silence (Morrison and Milliken, 2000). Many participants reported a scarcity of opportunities to communicate issues with management, attributing this to a restrictive communication culture and exclusion from decision-making processes. Prior research indicates that dissatisfaction with administrative communication leads to adverse organizational outcomes, including diminished institutional commitment, heightened burnout, and increased turnover rates (Xu and Farris, 2024). One participant stated:

The meeting agendas, discussions, everything is passive. At the end, they always ask if we have any questions. When such a restricted environment already exists, how could a person dare to speak? I remember, in one of our meetings in the past, one faculty member raised an issue over favoritism. His life was made hell by the management since then. He had to quit his job in the end. There must be open communication where leaders have the patience and endurance to take criticism as a sign of improvement rather than taking it personally and hurting teachers.

Research clearly indicates that open communication at the workplace is crucial for fostering a conflict-free environment, maintaining high-quality working relationships and well-being of educators (Adu-Oppong and Agyin-Birikorang, 2014). Since employees spend approximately one-third of their lives engaging with work responsibilities, they deserve a supportive environment where their concerns are heard, issues are resolved, and they are included in the decision-making process. When employees are excluded from decisions that affect them, it can lead to feelings of disempowerment and a lack of voice, thereby hindering the communication of issues (McPherson et al., 2022; Somech, 2006). The failure to cultivate such an environment inevitably leads to reduced motivation, often resulting in the decision to leave the profession. Another participant hassled,

There is a gap between the administration and teaching staff. We are not included in decision-making. Often, decisions are taken by the higher authorities but they make us sign the document. Teachers are allotted training and other duties without communication or consent. Sometimes, we are treated inhumanely. Once, I wanted to go to another city to meet my family due to their health issues, but I was stopped and, without understanding the situation, given a threat to either continue the job or quit. In such circumstances, quitting is always better than suffocating.

Some participants emphasized that there is no community of practice or organized activities where teachers can exchange ideas and collaborate on creative projects. Additionally, communication between teachers, students and parents is challenging due to restrictions imposed by management. It is widely recognized that children’s educational outcomes are maximized when teachers and parents establish collaborative partnerships rooted in constructive, transparent communication (Chen and Rivera-Vernazza, 2023). To ensure healthy relationships, open communication in education must be comprehensive, involving not only colleagues and the administration but also crucial stakeholders like students and parents. One participant stated:

“We are in this profession to support students. Many students are not expressive, but they sometimes want to share their concerns with us. However, we are not allowed to talk with students and parents. This barrier in communication sometimes makes us feel helpless."

These narratives make it clear that communication gaps and an inability to participate in decision-making processes have had a severe impact on teachers’ well-being. Research indicates that positive communication with school leadership can greatly improve teacher retention, reducing the likelihood of teachers leaving the profession, and can contribute to improved psychological and physical well-being (Ghamrawi et al., 2023; Pagán-Castaño et al., 2021; Ronfeldt and McQueen, 2017).

The absence of open communication, coupled with a dictatorial environment, is consistently recognized as toxic and detrimental to psychological well-being. Across the globe, toxic workplace environments are a pervasive issue with severe consequences. Such negativity erodes the well-being of individual employees, fostering mental health challenges, and significantly hampers the organization’s health through lost productivity, elevated staff turnover and a decline in overall performance (George, 2023). Some teachers felt that the culture of their workplace was toxic. Many teachers spoke negatively about the management because they felt they were not being listened to. This has caused further problems and increased animosity toward the management among many faculty members. One of the participants said:

I hate the management who want us to zip our mouths. I dislike such management where we want our institution to grow but our feedback is taken as an attack. This domineering nature and negative attitude deter many of us from sharing our concerns with the management. Instead, we often share our grievances with friends outside the workplace and sometimes on social media as anonymous chatter.

The lack of open communication channels and the fear of negative repercussions severely hinder teachers’ ability to express their grievances. This restrictive environment not only affects their well-being but also impacts their professional effectiveness.

#### 4.1.3 Professional jealously and toxic work environment

Vecchio (2000) defines employee jealousy as a pattern of thoughts, emotions and behaviors stemming from an employee’s actual or perceived loss of self-esteem or valued work-related outcomes. This loss, or the threat of it, is triggered by the perceived intrusion of a rival who could diminish one’s self-worth or undermine a significant professional relationship. Professional jealousy arises when colleagues resent others for the opportunities and recognition they receive. A good number of teachers highlighted this as one of the main factors that has severely affected their well-being. One teacher shared her perspective on this issue:

If I am getting the opportunity to visit other countries and represent my school, it is because of my efforts and the additional work that goes beyond my standard duties. Why should they prevent me from going? If they are not competent and I am, they should appreciate my professional development rather than hindering it and stopping me from enhancing my profile.

Professional jealousy is noted to exist not only among colleagues of equal status but also sometimes originates from higher management when superiors feel insecure or threatened by a subordinate’s superior capabilities or performance (Yu et al., 2025). Participant echoed these thoughts as,

You will find monsters and toxicity everywhere at my workplace. My school leader is jealous of my intellectual growth. I am effectively balancing my professional and academic journey. Recently, when I decided to apply for a PhD, my supervisor began giving me a hard time and adding extra workload to make it difficult for me to manage both. This pressure seems intended to force me to quit either my job or my studies. Such a harassing environment is toxic to my well-being.

Some participants believed that fostering a sense of unity and celebrating each other’s achievements could build resilience and create a positive teaching community. However, this would not be possible unless the management changed its attitude. One participant shared how his future had been ruined by the management, which had had an adverse effect on his well-being.

I had an opportunity to complete my studies abroad. Initially, my principal sidelined my leave application. Later, when the embassy interview deadline passed, I was given a letter of no objection. What is the use of such a letter after jeopardizing my future? It’s such a shame.

Clearly, professional jealousy creates significant obstacles to the academic and professional growth of employees. Moreover, in such a toxic environment, certain individuals may thrive despite lacking competence, succeeding instead through unethical flattery and by manipulating management (Westphal and Stern, 2006). Witnessing the progress of these undeserving individuals severely diminishes the motivation of deserving candidates, leading to widespread frustration and negatively impacting overall mental well-being. Another participant shared,

Teaching is a sacred profession, and our primary responsibility is to serve learners. However, at our workplace, talent is not respected. If you are skilled at flattering, pleasing the boss, and praising them, you are more likely to be promoted. I have been eligible to become a principal for 10 years, but because I do not engage in building false rapport with my superiors, I have never been given priority.

Workplace toxicity is further intensified when the recognition due to a deserving candidate is appropriated by superiors or credited to an individual who made no contribution. Such an environment, where hard work is not acknowledged or worse, is misattributed significantly increases anxiety and negatively impacts mental well-being (Alahiane et al., 2023). One more participant added how her ideas were exploited,

I am completely upset because my creative ideas were taken by the school Principal. Although I wasn’t seeking recognition, attributing these ideas to the principal and seeking the spotlight is an affront to teachers' creativity and dignity. My colleague was also misused by the principal several times. She was asked to write stories and chapters for books and publish them by adding the name of the principal as the first author. We are quiet doesn’t mean we are weak…

Professional jealousy and toxic work environment can be either episodic or consistent and arises from a range of factors. One study found that envy in the workplace negatively affects employee productivity (Manna, 2016). Meta-analytic reviews have identified key predictors of envy in the workplace, including individual differences, competition, negative behaviors and emotions, moral disengagement and social desirability (Li et al., 2023). Furthermore, jealousy may stem from leadership incompetence, personal failures and other individual factors (Bayar and Koca, 2021; Liu et al., 2021). In education, it often stems from a lack of trust, ongoing struggles for power and position, and perceived inequities in opportunities and recognition. Such dynamics can foster resentment and competition among colleagues, thereby impacting their professional relationships and the overall work environment.

### 4.2 Theme 2: barriers to sharing mental health issues

Mental health issues are an increasing concern, yet they are often overlooked in public discussion due to factors such as embarrassment, shame, concerns about privacy, and fear of discrimination (Choudhary et al., 2024). This reluctance is exacerbated by the common misconception that people with mental health conditions are dangerous or aggressive, which increases social distance (Lauber and Rössler, 2007). Consequently, access to care is often hindered by skepticism toward mental health services and available treatments (Choudhary et al., 2024). Pervasive stigma from family members, coupled with societal disapproval and devaluation of families affected by mental illness, further exacerbates the issue (Lauber and Rössler, 2007). In most South Asian countries, disclosing personal struggles is often considered shameful and a sign of weakness, leading individuals to avoid seeking help for mental health conditions in order to avoid prejudice and negative social repercussions. One of the studies underlined, “Within the South Asian culture, lies a social stigma toward mental health by a set of cultural norms and values, rejecting vulnerability and emotions that exhibit weakness, thus creating a barrier to retrieving psychological help” (Uddin, 2017, p. 4). Teachers in South Asia face many barriers to sharing mental health issues, primarily due to factors related to their workplace.

Participants were asked to identify the barriers that most often prevent them from seeking mental health support as teachers. The majority expressed that lack of expression, communication issues, and societal prejudices are the biggest obstacles. One participant stated,

As a female, if I talk about my mental health challenges with someone at work or in my social circle, people would easily label me as mentally ill, which could lead to serious consequences, such as reduced chances of getting married. To preserve my dignity, I prefer to remain silent.

Mental health is often overlooked because of its elusive nature; since it is not visible or measurable on a physical scale, people who disclose their struggles may face stigma (Corrigan et al., 2005). This often results in them being marginalized as either weak or exaggerating their issues, thus failing to receive necessary support or recommendations for medical treatment. Participant articulated,

No one wants to talk about mental health because this culture does not exist in our society. Internal battles and their harsh consequences cause people to suffer silently, but communicating struggles and seeking help are not priorities for most people.

Asian men are more vulnerable to mental illness due to the cultural perception that it prevents them from meeting the standards of ideal masculinity, preserving family reputation (‘saving face’), and fulfilling their moral obligation to provide for others (Livingston et al., 2018). One of the male participants highlighted that discussing mental health is more challenging for men than for women due to traditional notions of masculinity and the expected role of the dominant figure in South Asian society.

Who would believe that I face regular setbacks at work? I want to cry, vent my frustrations, and talk about how cruelly my workplace uses me. But who would believe me? Society expects men to be strong and conceal their true feelings. I am a teacher, and I am human—yet will society allow us to express ourselves openly?

SAARC countries are currently contending with severe economic and social crises, characterized by high inflation and limited job opportunities (Zeb et al., 2022), pushing citizens toward a “survival of the fittest” mentality. In a highly competitive job market, where multiple applications compete for a single position, admitting to struggles with mental health is considered a significant risk. Consequently, many individuals, prioritizing the need to secure income and “bring food to the table,” choose to remain silent and do not disclose their personal mental health struggles despite the difficulties they face. Few participants fear negative consequences such as professional repercussions or judgment from colleagues and students, which prevent them from sharing mental health challenges with others:

I have been in the field of teaching for 20 years, and I have experienced multiple episodes of mental health issues at work. The reason I never shared these experiences with anyone is that I fear being judged by my students and colleagues. We need to strengthen the trust system and normalize the discussion of mental health issues as a regular part of life.

Mental health challenges are widespread, and they are particularly prevalent in academia. Both students and educators experience difficulties, but often suffer in silence due to the associated stigma (Müller, 2020). According to many participants, there is a lack of awareness about mental health across academia, which has deterred them from seeking support when needed. They noted that many teachers would rather quit their jobs and face personal hardship than seek help. This is either because they do not know how or whom to approach, or because they are unsure whether their internal struggles constitute a mental health issue due to a lack of awareness. Participants highlighted that there is no concept of counselors or psychologists to support teachers’ well-being because the management believes that teachers do not face mental health challenges in the workplace. If they face such challenges, the management assumes that teachers are empowered to tackle these issues independently. One of the participants highlighted multiple issues:

Not surprisingly, the greatest barrier to quality professional development in fragile contexts is the difficult conditions in which teachers work. The lack of remuneration, overcrowded classrooms, the potential for sexual harassment or abuse, a lack of respect from school leaders and community members, violence in school, too many needy students, and a lack of teaching and learning materials, all contribute to such difficult working conditions. The culture of availability of mental health services at the workplace for teachers' wellbeing does not exist, and it is a shame that we have to witness and fight our battles independently.

Our extensive experience of working in the field of teacher education confirms that there are no formal courses on mental health and well-being in teacher education programs in the selected countries which is also confirmed by many research studies (Nishio et al., 2020; Nguyen et al., 2020; Zhou et al., 2019). When asked whether they had attended workshops or counseling sessions, all participants indicated that they had not. The main barriers to participants sharing mental health issues with others are a lack of culture surrounding mental health, a lack of awareness, and a lack of mental health services for teachers at work. Notably, analysis of the data from the from Pakistan and Afghanistan reported a higher incidence of these challenges, yet they also demonstrated the least engagement with coping strategies. Additionally, participants from Pakistan, Afghanistan, Bhutan, and the Maldives faced greater challenges in expressing their struggles compared to those from other countries.

### 4.3 Theme 3: coping strategies

In order to answer the research question regarding the most effective practices and strategies for addressing mental health issues, responses were collected and categorized under Theme 3. When asked which strategies are more effective for dealing with mental health challenges in the workplace, most participants said that they do not use any strategies; instead, they take on the burden themselves and feel overwhelmed by workplace issues. However, a few participants mentioned that they usually share their concerns with friends. A key takeaway from the work of Braun and Lascelles (2024) is that having a strong support system is vital for mental wellbeing. They emphasize that confiding in family and friends can effectively reduce stress and enhance mental well-being.

One participant said:

I always share my well-being issues with my husband. He is very supportive and always gives me good advice to help me manage mental health issues and stress.

A good number of participants expressed that they trust family members for taking guidance and counseling in times of need. One of them stressed,

I entered this profession because of my parent's wish. I love this profession. Sometimes, the situation forces me to quit but it is due to my parental involvement in counseling me with day-to-day teaching challenges, I am growing intellectually and grooming my students morally.

In South Asian cultures, especially men often refrain from sharing personal struggles, particularly those related to mental health or workplace challenges, with their immediate family to avoid causing stress or creating a strained home environment (Joshi and Krishnan, 2025). In these situations, friends frequently step in to serve as crucial listeners and sources of support (Khan and Sultan, 2023). One of the participants added:

My friends are the best advisors. They always guide me on what is beneficial and what is not. They are consistently supportive, and I believe that having a strong support system makes me feel more confident in effectively handling any challenges.

Another participant remarked,

Talking with colleagues and experts helps me. Sometimes it means a lot to know that others have the same or similar problems because then we know that we are not alone.

Several research studies have highlighted the strategies commonly employed by teachers to promote their own well-being. These include relaxation or meditation practices, positive reframing, active coping and planning (Agyapong et al., 2023). Furthermore, problem-solving, exercise and pursuing hobbies, getting sufficient sleep have been identified as effective methods of enhancing emotional well-being (Braun-Lewensohn, 2016; Corbett et al., 2022; Emeljanovas et al., 2023; Rajesh et al., 2022). Several participants emphasized the importance of recreational activities in coping with mental health challenges in the workplace. They highlighted walking, yoga, meditation, art, music, breathing techniques, naps and coloring as effective strategies. Another group emphasized the importance of attending workshops and completing tasks efficiently at work. Palmerin (2023) recommends that attending self-care and grooming workshops can be an effective way to manage mental health challenges. These diverse approaches collectively contribute to stress management and enhance overall well-being in the work environment. Some participants emphasized the importance of effective communication in resolving disputes with management or students. Studies consistently show that positive communication between teachers and school leadership leads to better psychological and physical well-being. This is a finding supported by the research of Ghamrawi et al. (2023); Pagán-Castaño et al. (2021) and Ronfeldt and McQueen (2017).

I think my turning point was when I had an open communication with the principal regarding physical and mental health due to pressure. We worked out differences and try our level best to manage. Secondly, I use breathing exercises for stress management. I also kept on training sessions how to improve my teaching and communication skills with the students.

Seeking solutions for personal struggles through media and online resources offers an important, non-physical strategy for finding help (Yuen et al., 2025). However, this approach critically requires strong screening techniques to discern factual information from misinformation. Despite this necessary caution, utilizing online resources or media remains a viable way to find support, as articulated by one participant,

Whenever I come across a mental stress situation, I always reflect and try to figure out the possible reasons. I have always been an obedient student throughout my life, and I respect everybody around me. I try to read what experts say about this. A few years ago, I studied classroom management techniques, and recently, I studied teachers' styles. I try to adapt those and change my approach and behavior.

While a lack of open communication is known to cause frustration and negatively affect teachers’ mental health as mentioned previously and by researchers Xu and Farris (2024), participants suggest that being open with stakeholders and offering students chances for open discussion can promote a friendly environment and help prevent these mental health issues. While promoting openness, teachers must establish a clear boundary of respect. Caution is necessary; becoming overly friendly can backfire, resulting in teachers losing respect and students engaging in misbehavior. Participant mentioned,

What I found effective was to fill the communication gap. I tried to listen to what was going on in the school and with those students, especially how they were being treated. I gathered them all and had a good, long conversation. They poured their hearts out to me. They were being mistreated and were labeled by teachers, but few teachers also faced disrespect like me. What I found was that students took the wrong meaning from my words, and they were supported by a group of new staff members.

Furthermore, some participants expressed that maintaining open communication is a valuable method for strengthening connections among stakeholders and cultivating more favorable relationships as suggested by Chen and Rivera-Vernazza (2023). Another participant shared:

An effective strategy I use is paying attention to my needs and communicating them as often as possible with the management and my students. I believe in embracing vulnerability and sharing narratives, as these practices help strengthen the bond between teachers, staff, administration, and students.

Open communication is not solely about providing opportunities for others to express themselves; it is also a reciprocal process. Listeners benefit significantly by hearing diverse narratives, which fosters empathy, allows for connection with shared experiences, and enables the exchange of common thoughts and sources of pleasure (Kluger and Itzchakov, 2022). One participant articulated this as:

Taking people to the level where they feel comfortable to discuss their issues, taking them swiftly into the environment where thoughts other than their stress creators always prove to help utilize positive capacities of people - either students or colleagues. Sitting and talking about old friends, old pictures, recalling their pleasant memories, and change of environment by travel seem the most effective strategies in this regard that I often use.

A few participants believe that reading the narratives and stories of other teachers, as well as sharing their own experiences, can lead to significant change. They feel that the teaching community can learn a great deal from one another and more effectively manage challenges. One participant said:

I talk a lot with my students and try to write articles about classroom issues and then find relevant videos to share on social platforms where teachers can view them. By educating them through these resources, I aim to help address and solve these problems.

Another strategy that most participants highlighted was community engagement. Researchers have also identified community engagement as a buffer against stress (Latif et al., 2022). It enables participants to spend quality time with marginalized individuals, share their expertise and learn from others in a supportive environment. One participant explained:

Whenever I have free time, which is quite rare, I spend it doing community work with marginalized people. What I learn from them is to love, spread positivity, avoid jealousy, treat others with kindness, have patience, and thank God for making me a teacher.

Carmola Hauf and Bond (2002) argue that to create effective mental health prevention and promotion initiatives, it is crucial to involve and collaborate with many different stakeholders. This collaborative approach ensures that programs are well-balanced and include diverse perspectives, which is a key characteristic of successful initiatives (Carmola Hauf and Bond, 2002). Establishing healthy boundaries, asking for support, problem focused strategies, practicing time management and problem-solving skills are some other strategies identified by the participants. Beyond their roles and responsibilities, teachers are expected to put aside their ideologies and differences when teaching learners. This requires greater tolerance, acceptance, respect, patience and understanding.

When asked how they maintain their motivation and engagement at work, participants shared a variety of techniques that motivate them. For most participants, motivation stems from teamwork (Ananda and Eriza, 2023), the positive energy of children, and the desire to impart knowledge, skills, and learning strategies. One of the participants reported,

We all work together as a team on such children both individually and together with classes, maintaining motivation is taking into account the best interests of the child and expecting that the child will develop into a full-fledged person and engage in good deeds in the future. My inner motivation and energy are contagious to those around me, and together everything works out well.

Some participants shared that reading books maintain their motivation. Levine et al. (2022) also emphasized that recreational reading reduces psychological stress and associated with the autonomous motivation. One of them said,

I try to keep myself away from stressing scenario and thoughts and that can be best done by reading a good book or a travelogue.

A good number of participants were in view that setting clear goals, and acceptance toward learning from failure are crucial to stay focused and motivated.

I always break down goals into smaller ones and celebrate my progress along the way. Instead of being discouraged by failures, I see them as opportunities to learn and achieve goals.

This analysis clearly identifies workplace stressors contributing to burnout and mental health issues. Furthermore, it highlights the potential reasons why teachers, despite their suffering, often hesitate to share their issues or seek professional help. Uniquely, the study also provides information on small-scale, individual well-being strategies currently used by teachers in these countries. This shared information can subsequently be utilized to develop large-scale professional training programs aimed at improving teacher well-being.

## 5 Discussion

Mental health and well-being are among the most crucial yet often overlooked areas in academia (Müller, 2020). This neglect can have significant consequences for both educators and students, impacting overall educational outcomes and personal development (George, 2023). It is crucial to understand the mental health challenges faced by teachers and the current practices they use to support their personal well-being, as multiple studies have reported that teachers with mental health issues who do not receive adequate support may experience decreased productivity, reduced self-efficacy, increased absenteeism and higher stress levels, which can ultimately affect the quality of education they provide (Ferguson et al., 2022; George, 2023; Kundu and Bej, 2024; Maslach and Leiter, 2016).

This study collected narratives from teachers in South Asian SAARC countries to understand the mental health challenges they face, the barriers preventing them from sharing these issues with others, and their strategies for addressing these challenges. The initial themes clearly indicate that teacher burnout stems from a combination of systemic factors. In the South Asian context, an excessive workload characterized by unsustainable demands, often compounded by a high volume of uncompensated administrative and non-instructional tasks, contributes significantly to burnout and diminished mental well-being. These findings are consistent with previous research that has identified such stressors as the main cause of mental health issues among educators (Andina-Díaz et al., 2025; Pressley et al., 2026; Schaack et al., 2022; Siddiqui et al., 2023a). Participants also identified ethical dilemmas as a significant source of frustration, particularly with regard to the pressure to provide undue support to ineligible students. This creates a moral conflict that directly impacts mental well-being. Indeed, when systemic pressures prevent educators from acting in accordance with their professional ethics, the resulting conflict can lead to chronic burnout (Oberg, 2025). Furthermore, economic inflation in SAARC countries (Zeb et al., 2022) means that many people must balance a teaching career with their own studies in order to remain financially sustainable (Russell et al., 2025). These student teachers’ excessive workload also negatively affects their mental well-being due to the high demands placed on them. Adding to these stressors, teachers are often required to manage inclusive classrooms without adequate training. This lack of preparation can leave them feeling incompetent and inadequate, further fuelling frustration and burnout (Alhassan et al., 2025; Candeias et al., 2021; Kazmi et al., 2023; Singal, 2021). Ultimately, these high demands frequently cause educators to leave the profession (Amitai and Van Houtte, 2022; Arnold and Rahimi, 2025; Benevene et al., 2020; Siddiqui et al., 2023a), thereby exacerbating the ongoing shortage of teachers in academia. Ineffective communication with management and stakeholders emerged as another critical factor influencing educator well-being. Participants noted that a lack of safe spaces to share concerns without retaliation hinders professional support (Xu and Farris, 2024). This frustration is compounded by poor interpersonal communication between colleagues and parents. Ultimately, when educators are sidelined during decision-making, they feel unheard and disempowered (McPherson et al., 2022), leading to a significant decline in their psychological health (Ghamrawi et al., 2023). Excessive workloads and poor communication are not the only factors that compromise employee mental well-being; professional jealousy and interpersonal friction also play a role. Participants described a culture in which hurdles are intentionally created to sabotage colleagues’ success, and in which undue recognition and support are provided to ineligible candidates. Such practices foster a toxic environment that severely impacts morale, as Alahiane et al. (2023) have identified as a significant contributor to psychological distress in the workplace. Crucially, the problems affecting teacher mental well-being and retention, as observed in this study, are similar to those documented in developed nations, suggesting these issues are universal and not confined solely to the SAARC region (Amitai and Van Houtte, 2022; Schaack et al., 2022; Wiggan et al., 2021). These factors have also been identified as predictors of mental health issues among educators in many international studies as well (Agyapong et al., 2022; Bayar and Koca, 2021; Ghamrawi et al., 2023; Madigan et al., 2023).

Second theme of the study identifies cultural stigma and limited awareness as the chief barriers to mental health transparency. Lauber and Rössler (2007) and Müller (2020) clearly expressed that educator experience difficulties but often suffer in silence due to associated stigma, prejudice and disapproval from society, where people starting to socially distance themselves from people showing signs of mental illness. In addition, revealing mental health struggles is seen as dangerous: a perceived weakness that may lead to exclusion from job consideration. According to Kabir et al. (2024), the pervasive stigma surrounding mental health in South Asia ensures it remains a low-priority issue. For teachers, this environment is particularly damaging; the combination of social shame and the high cost of professional care ensures that their mental health concerns remain unaddressed and unexpressed Therefore, to ensure financial stability, many people suppress their mental health issues and choose not to disclose their personal struggles.

Theme three identifies practical strategies and adaptive solutions for managing mental health issues despite limited institutional resources. Participants highlighted that they often rely on various strategies to cope with mental health challenges. These strategies include sharing feelings with friends and family. While expressing emotions fosters self-awareness and cathartic relief, active listening cultivates deep empathy and trust and together, these processes strengthen social support systems and human connection (Kluger and Itzchakov, 2022). The other strategies included practicing meditation, engaging in recreational activities, taking care of physical fitness, improving communication, participating in community engagement, reading and establishing healthy boundaries. While these strategies are beneficial, participants also emphasized the importance of acknowledging mental health challenges and maintaining the determination to address them effectively. These strategies are in line with international literature and have been shown to be effective in promoting wellbeing (Agyapong et al., 2023; Braun and Lascelles, 2024; Braun-Lewensohn, 2016; Corbett et al., 2022; Emeljanovas et al., 2023; Ghamrawi et al., 2023; Latif et al., 2022; Levine et al., 2022; Rajesh et al., 2022).

A key finding of this research is that almost every participant reported experiencing mental health challenges. The WHO’s South-East Asia Region is experiencing a significant mental health crisis, yet the policies designed to address this issue are largely ineffective (Sharan et al., 2017). Studies have shown that although many countries in the region have national mental health policies, these often lack sufficient funding and effective implementation, as well as legal protections for people with mental health conditions (Mudunna et al., 2025; Sharan et al., 2017). This systemic failure highlights a significant policy gap, particularly with regard to targeted support for teachers’ mental well-being.

## 6 Conclusion

The findings of this study provide a baseline for understanding the complex mental health issues faced by educators in South Asia. By adopting a ‘teacher-centered’ approach, this study makes a significant and rare contribution to the literature, filling an important empirical gap in these developing nations. Beyond merely documenting distress and possible reasons for mental health issues, this research also highlights how teachers have developed informal, resourceful coping strategies, such as peer support and seeking help online. However, these efforts exist in isolation and systemic support from policymakers and educational institutions is virtually non-existent. This shifts the narrative from individual resilience to demanding structural reform, proving that personal coping is a temporary solution, not a long-term substitute for institutional intervention. The summary of the themes is found in Table 2.

**Table 2:** Summary of the themes generated with main points.

Main theme	Sub themes/points discussed	
Common mental health challenge encountered by teachers	Excessive workload and burnout	Unmanageable workload Unexpected additional demands Poor work life balance Misuse (under or over use of talent) Incompetent Leadership Lack of Training and Resources Uncompetitive salaries Poor remuneration
	Lack of communication and exclusion from decision making	Leaders’ inability to accept constructive criticism Exclusion of teachers from decision-making processes Poor cross-functional teamwork Inadequate platforms for transparent dialog Fear of reprisal for open communication
	Professional jealously and toxic work environment	Stagnation of talent growth Absence of acknowledgment and gratitude Lack of recognition and appreciation Favoritism based on insincere flattery
Barriers to sharing mental health issues	Stigma associated with mental illness Challenges in forming marital relationships Limited understanding of mental health conditions Perceived threat to masculinity Adverse career consequences Negative peer judgment and prejudice Insufficient institutional and social support Shortage of qualified counselors and practitioners	
Effective practices and coping strategies	Seeking support and advice from family and friends Meditation Practices Practicing relaxation techniques Positive reframing and proactive planning Regular physical exercise Applying problem-solving techniques Engagement with creative hobbies (Art, Music) Prioritizing physical well-being (e.g., yoga, walking, adequate sleep) Investing in personal growth and skill development (e.g., workshops, reading, writing, time management, fostering self-motivation and self-belief) Fostering effective communication with colleagues, authorities, and students Engaging with the community and sharing experiences Exploring through travel	

## 7 Limitations and strengths of the study

A primary limitation of this study is the inability to disclose the specific nationalities of individual participants. This restriction stems from the informed consent and ethical agreements established during the data collection phase, which preclude the inclusion of country-specific contextual data or the development of nation-specific policy recommendations. Consequently, while the emerging themes showed a high degree of cross-national commonality among all participants, this constraint prevented the exploration of nuanced cross-cultural differences. We therefore recommend that future research is dedicated to exploring these specific cultural variations and developing tailored recommendations for each country. The findings can be generalized within the context of seven South Asian countries in a school setting only. To enhance the generalizability of these findings, future research should expand its scope to include educators from the higher education and vocational training sectors. Incorporating a broader demographic would provide a more comprehensive understanding of the mental health challenges faced across the entire academic spectrum, and reveal how institutional pressures vary between secondary and tertiary levels. Additionally, there is a sampling limitation, as only school teachers were considered. More females than male participated in the study, highlighting the gender gap. The study did not differentiate between public and private school teachers, which represents a potential gap. To enhance the generalizability of these findings, future studies should adopt a stratified sampling approach that encompasses a more diverse demographic profile. This should include educators from public and private institutions in urban, rural and suburban areas. Regarding methodological limitations, the study explored the phenomena qualitatively and did not include a quantitative analysis. The selection of participants had notable limitations, primarily due to the reliance on a single online platform (Facebook), which introduced significant bias. This method likely yielded a non-representative sample by excluding teachers from remote regions, those unfamiliar with this particular global forum, and those who are not tech-savvy. This undermines the qualitative goal of capturing a universally experienced ‘essence’ across South Asian contexts. Furthermore, to mitigate the ‘digital divide’ and ensure the inclusion of participants with limited internet connectivity, researchers should leverage the grassroots networks of local universities and NGOs to facilitate data collection in underrepresented regions. Furthermore, the requirement of 10 years’ teaching experience ensures seniority, but does not inherently guarantee that participants have the deepest or most relevant lived experience concerning the specific phenomenon being explored. It is also acknowledged that the study’s exclusive reliance on manual analysis, without the use of qualitative data analysis software, is a limitation.

This study is however, particularly strong because it is a unique and large-scale project that collected extensive data from countries with similar geographical and economic conditions. A key benefit of this research is that it not only identifies the causes of mental health issues but also explores practical solutions used by educators themselves.

## 8 Recommendations

To address the challenges teachers, face with mental health, it is crucial to normalize these issues and encourage open communication to eliminate the stigma surrounding it. Awareness of mental health should be integrated into the regular practices of each institution not only for teachers but also the leadership. Policies on institutional mental health should be developed in consultation with teachers to support their well-being. Establishing a mental health support unit within the workplace is essential. To create a supportive work environment, management should involve teachers in decision-making, and handle criticism constructively. Additionally, engaging teachers in reflective practices, fostering a sense of ownership, and helping them understand their teaching identity are important steps. Furthermore, it is highly recommended that teacher certification and degree programs incorporate wellbeing modules. These courses should be designed to strengthen the professional identity of educators while equipping them to nurture the personal development of their students.

Addressing the challenges faced by teachers not only helps improve teachers’ overall well-being but also contributes to a healthier, more supportive work environment. By recognizing and supporting their mental health needs, schools can enhance teacher retention, foster a positive workplace culture, and ensure that teachers are better equipped to support their students’ needs effectively.

## Data Availability

The raw data supporting the conclusions of this article will be made available by the authors, without undue reservation.

## Footnotes

1
https://www.facebook.com/GlobalTeacherForum
