# Supplementary material for: Exploring mental health challenges encountered by educators in the South Asian countries—a qualitative survey
Source: Front Psychol. 2026 Apr 30;17:1682410. doi: 10.3389/fpsyg.2026.1682410 (PMC13171593; doi:10.3389/fpsyg.2026.1682410)
Supplement: Supplementary file 1 [file Supplementary_file_1.docx]

Supplementary File

**Appendix 1**

**ONLINE CONSENT FORM**

You are invited to participate in a research study exploring the mental health challenges faced by experienced educators across the SAARC region.

The goal of this research is to gain a broad vision of mental health issues among school teachers in South Asian countries. By collecting insights, we aim to identify common stressors and systemic challenges to better support teacher well-being.

To participate in this study, you must:

- Must be a volunteer member of Global Forum for Teacher Educators
- Be a resident of any one country: Pakistan, India, Sri Lanka, the Maldives, Bhutan, Afghanistan and Nepal.
- Have a minimum of ten years of teaching experience.
- Be currently employed at a public or private school.

If you agree to participate, the process will involve:

A short introductory briefing by the lead researcher regarding study objectives and protocols. It will be followed by a recorded online interview via Zoom consisting of three semi-structured questions. This will take approximately 20 minutes. You will be asked to provide verbal consent at the start of the recorded session.

All data will remain confidential. Your name, specific school or nationality will not be mentioned in any reports or publications. Interview recordings and transcripts will be stored on a password-protected drive, accessible only to the research team. Recordings will be transcribed by the research team for analytical purposes.

Your participation is entirely voluntary. You have the right to refuse to answer any specific question; withdraw from the study at any time during the interview without providing a reason and without any negative consequences. There are no physical or psychological risks associated with this study.

By clicking **I Agree** below, you confirm that

- I have read and understood the information provided above.
- I meet the eligibility criteria.
- I understand that my interview will be recorded and transcribed.
- I voluntarily agree to participate in this study.

[ ] **I Agree** [ ] **I Do Not Agree**

*(Proceed to demographics information)*

**Appendix 2**

**VERBAL CONSENT FORM**

Dear participants,

I would like to thank you for taking the time to participate in today’s interview. The aim of this research is to gain an understanding of the mental health issues faced by school teachers in South Asian countries. Your participation is crucial to understanding and addressing these challenges. The interview will take approximately 20 minutes. You can stop at any time, and if you wish to exit the interview voluntarily, you can do so. There are no known physical or psychological risks associated with participating in this study. The interview will be recorded, but only our team will have access to the research records. Do you understand the procedure and give your consent for your interview to be recorded? Do you have any questions before we begin?
